# High-throughput STELA provides a rapid test for the diagnosis of telomere biology disorders

**DOI:** 10.1007/s00439-021-02257-4

**Published:** 2021-03-11

**Authors:** Kevin Norris, Amanda J. Walne, Mark J. Ponsford, Kez Cleal, Julia W. Grimstead, Alicia Ellison, Jenna Alnajar, Inderjeet Dokal, Tom Vulliamy, Duncan M. Baird

**Affiliations:** 1 Division of Cancer and Genetics, School of Medicine, Cardiff University, Heath Park, Cardiff, CF14 4XN UK; 2 Centre for Genomics and Child Health, Blizard Institute, Barts and The London School of Medicine and Dentistry, Queen Mary University of London, London, E1 2AT UK; 3 Immunodeficiency Centre for Wales, University Hospital of Wales, Heath Park, Cardiff, CF14 4XW UK; 4 Division of Infection, Inflammation and Immunity, School of Medicine, Cardiff University, Heath Park, Cardiff, CF14 4XN UK

## Abstract

Telomere biology disorders are complex clinical conditions that arise due to mutations in genes required for telomere maintenance. Telomere length has been utilised as part of the diagnostic work-up of patients with these diseases; here, we have tested the utility of high-throughput STELA (HT-STELA) for this purpose. HT-STELA was applied to a cohort of unaffected individuals (*n* = 171) and a retrospective cohort of mutation carriers (*n* = 172). HT-STELA displayed a low measurement error with inter- and intra-assay coefficient of variance of 2.3% and 1.8%, respectively. Whilst telomere length in unaffected individuals declined as a function of age, telomere length in mutation carriers appeared to increase due to a preponderance of shorter telomeres detected in younger individuals (< 20 years of age). These individuals were more severely affected, and age-adjusted telomere length differentials could be used to stratify the cohort for overall survival (Hazard Ratio = 5.6 (1.5–20.5); *p* < 0.0001). Telomere lengths of asymptomatic mutation carriers were shorter than controls (*p* < 0.0001), but longer than symptomatic mutation carriers (*p* < 0.0001) and telomere length heterogeneity was dependent on the diagnosis and mutational status. Our data show that the ability of HT-STELA to detect short telomere lengths, that are not readily detected with other methods, means it can provide powerful diagnostic discrimination and prognostic information. The rapid format, with a low measurement error, demonstrates that HT-STELA is a new high-quality laboratory test for the clinical diagnosis of an underlying telomeropathy.

**Supplementary Information:**

The online version contains supplementary material available at 10.1007/s00439-021-02257-4.

## Introduction

The first connection between bone marrow failure and defective telomere maintenance came when the *DKC1* gene, responsible for the X-linked form of dyskeratosis congenita (DC), was shown to encode a component of telomerase (Mitchell et al. 1999). Subsequent identification of other genes causing autosomal dominant and recessive forms of this disease firmly established DC as a clinical manifestation of compromised telomere function in humans (Armanios et al. 2005; Savage et al. 2008; Vulliamy et al. 2001). Along with the increasing number of telomere-related genes found to cause DC (Dokal et al. 2015), there also came an expanding range of clinical manifestations associated with inherited defects in telomere biology. Most significantly, and most probably still under-diagnosed, are the later onset diseases including myelodysplasia as well as lung and liver fibrosis (Armanios et al. 2007; Calado et al. 2009; Marrone et al. 2007). Collectively, these conditions are referred to as telomeropathies.

Whilst the telomeropathies exhibit diverse clinical manifestations, they share the common molecular defect of short telomeres relative to unaffected individuals (Alder et al. 2008; Vulliamy et al. 2001; Walne et al. 2008). Telomere length in patients with telomeropathies have previously been examined using hybridisation-based methodologies including terminal restriction fragment (TRF) analysis and fluorescence in situ hybridisation (FISH) (Aubert et al. 2011). Telomeric FISH has been adapted for use with flow cytometry (flow-FISH) and provides relatively low throughput, but highly reproducible technique with a low measurement error and, as such, has been adapted as an industry standard for clinical telomere length measurements (Alder et al. 2018; Rufer et al. 1998). The telomere length distributions observed with TRF, FISH and flow-FISH are determined by the use of telomere repeat containing hybridisation probes (Lansdorp et al. 1996; Rufer et al. 1998), as shorter telomeres have less target sequences for the hybridisation probe the detectable signal decreases. These methodologies, therefore, exhibit a poorly defined lower length threshold, below which telomeres cannot be detected and, therefore, do not describe the full spectrum of telomere lengths in individual samples. Quantitative PCR-based methods have also been employed that facilitate the analysis of large numbers of samples; however, these lack linearity with other methods in the short telomere length range, provide no information on telomere length distributions and display a high measurement error that renders them less suitable for clinical applications (Gutierrez-Rodrigues et al. 2014; Khincha et al. 2017; Wang et al. 2018).

Single Telomere Length Analysis (STELA) is a high-resolution single-molecule approach to determine telomere length distributions including in the lower length ranges that are not so apparent with other commonly used technologies (Aubert et al. 2011; Baird et al. 2003; Britt-Compton et al. 2006). STELA is labour intensive and Southern hybridisation based and is, thus, not well suited for the analysis of large cohorts, or for clinical laboratory applications. To overcome these limitations STELA has been adapted for high-throughput analysis (HT-STELA) of cancer cell populations and successfully applied to predict response to treatment in patients with chronic lymphocytic leukaemia (Norris et al. 2019; Tissino et al. 2020). Here, we employed HT-STELA to examine the full extent of telomere erosion in individuals with dyskeratosis congenita and related disorders, and to determine the utility of high-resolution telomere length analysis as a potential diagnostic test for telomeropathies.

## Materials and methods

### Patient and control samples

The patients included in this study had all been recruited to the London Dyskeratosis Congenita Registry. 172 subjects from 134 unrelated families were selected on the basis that they had a potentially pathogenic variant in one of the genes known to cause DC and a DNA sample that passed quality control checks. There were 113 males and 59 females with a median age of 22 years and an age range of 0.5–77 years. Using previously described criteria (Vulliamy et al. 2006), we subdivided the patients into the following clinical subtypes: (1) those who fulfilled a clinical diagnosis of DC (*n* = 73); (2) those who resembled the Hoyeraal–Hreidarsson syndrome phenotype (HHS, *n* = 15); (3) those who had bone marrow failure and variable extra-haematological abnormalities that were insufficient for a clinical diagnosis of a known bone marrow failure syndrome (BMF, *n* = 47); (4) those who had liver and/or lung pathology reminiscent of a telomeropathy (LLD, *n* = 5) and (5) those who were asymptomatic carriers (ASM, *n* = 32). The breakdown of all samples according to the gene involved and the category of clinical presentation is shown in Table 1. All participants provided written informed consent in accordance with the declaration of Helsinki, with the approval of our local research ethics committee (London-City and East, reference 07/Q0603/5).

**Table 1:** Distribution of genes mutated and clinical phenotypes among 172 affected individuals

	ASM	BMF	DC	HHS	LLD	All
*ACD*	0	2	0	0	0	2
*DKC1*	2	1	44	5	0	52
*RTEL1*	0	2	0	0	0	2
*TERC*	17	16	9	2	2	46
*TERT*	13	21	10	3	3	50
*TINF2*	0	5	10	5	0	20
All	32	47	73	15	5	172

*ASM* asymptomatic, *BMF* bone marrow failure, *DC* dyskeratosis congenital, *HHS* Hoyeraal Hreidarsson syndrome, *LLD* liver and/or lung disease, *ACD* ACD shelterin complex subunit and telomerase recruitment factor (NM_001082486.1), *DKC1* dyskerin pseudouridine synthase 1 (NM_001363.4), *RTEL1* regulator of telomere elongation helicase 1 (NM_032957.4), *TERC* telomerase RNA component (NR_001566.1), *TERT* telomerase reverse transcriptase (NM_198253.2), *TINF2* TERF1 interacting nuclear factor 2 (NM_001099274.1)

Controls were recruited from spouses and unaffected sibs (excluding those in TERC and TERT families) of patients attending clinic and healthy volunteers. Control participants were screened at time of consent for past medical history and excluded if any personal history of recurrent or severe infections (> 2 oral antibiotic courses per year, or any admissions for intravenous antibiotics) suggestive of underlying immune deficiency. Additional healthy adult controls were also recruited in Cardiff with approval from the London Surrey Borders REC (reference 17/LO/0078).

### Variant detection

DNA samples were screened for potentially pathogenic variants in genes known to cause DC by a number of different methods over the years. Prior to the advent of next-generation sequencing, screening procedures included targeted PCR amplification followed by single-strand conformation polymorphism analysis or denaturing high performance liquid chromatography. More recently, samples have been screened using a bespoke gene panel containing genes known to cause a variety of inherited bone marrow failure syndromes. This was performed using a SureSelect™ library and library preparation kit (Agilent) prior to paired-end sequencing on a MiSeq platform (Illumina). Data from the sequencing runs were processed using an in-house pipeline involving the Burrows-Wheeler aligner, Samtools, variant calling with the Genome Analysis Toolkit and annotation with Annovar (Li and Durbin 2009; Li et al. 2009; Van der Auwera et al. 2013; Wang et al. 2010). Variants were filtered for a minor allele frequency of < 0.001 on the Exome Aggregation Consortium database. All variants have been validated by direct Sanger sequencing using BigDye™ Terminator Cycle Sequencing kits (Applied Biosystems, Source Biosciences), followed by capillary electrophoresis.

### Telomere length and fusion analysis

17p and allele-specific XpYp STELA were performed to determine telomere length according to standard protocols (Baird et al. 2003; Capper et al. 2007; Jones et al. 2014). Briefly, genomic DNA was isolated from peripheral blood leukocytes using the QIAamp-DNA Minikit (Qiagen) or the Gentra Puregene Blood kit (Qiagen) and diluted to 10 ng/μL in 10 mM Tris–HCl (pH 7.5). Ten nanograms of DNA was further diluted to 250 pg/μL in a volume of 40 μL containing 1 μM Telorette2 linker and 10 mM Tris–HCl (pH 7.5). 1μL of this DNA/Telorette 2 solution were subjected to PCR in a 10 μL reaction containing 0.5 μM telomere-specific primers, 0.5 μM Teltail primer and 0.5 U of a 10:1 mixture of Taq (Thermo Fisher Scientific) and Pwo polymerase (Roche). The PCR products were resolved by 0.5% TAE agarose gel electrophoresis and were detected by Southern hybridization with a random-primed α-^32^P-labelled (GE Healthcare) TTAGGG repeat probe. High-throughput STELA was performed as previously described (Norris et al. 2019; Tissino et al. 2020). Briefly, 40 ng (XpYp) or 20 ng (17p) of genomic DNA was added to triplicate 30 μL PCR reactions containing telomere-adjacent primers specific for the XpYp telomere (XpYpC: 5**ʹ** -CAGGGACCGGGACAAATAGAC-3**ʹ**) and the 17p telomere (17pseq1rev: 5**ʹ**-GAATCCACGGATTGCTTTGTGTAC-3**ʹ**) and 1.5 U of a 10:1 mixture of Taq (Thermo Fisher Scientific) and Pwo polymerase (Roche). Thermal cycling conditions were: 23 cycles of 94 °C for 20 s, 65 °C for 30 s, and 68 °C for 5 min. Amplified fragments were resolved using capillary gel electrophoresis and mean telomere length determined using PROSize software (Agilent).

Telomere fusion assays were performed as described (Capper et al. 2007; Letsolo et al. 2010; Tankimanova et al. 2012). Briefly, 50 ng of phenol/chloroform extracted DNA were subjected to PCR in a 10 μL reaction containing 0.5 μM telomere-adjacent oligonucleotide primers (XpYpM: 5**ʹ**-ACCAGGTTTTCCAGTGTGTT-3**ʹ**, 17p6: 5**ʹ**-GGCTGAACTATAGCCTCTGC-3**ʹ** and 21q1: 5**ʹ**-CTTGGTGTCGAGAGAGGTAG-3**ʹ**) and 0.5 U of a 10:1 mixture of Taq (Thermo Fisher Scientific) and Pwo polymerase (Roche). Fusion molecules were detected by Southern hybridisation as described above and detected with a 21q, XpYp or 17p telomere-adjacent probes (Letsolo et al. 2010).

### Statistical analysis

Statistical analysis was undertaken using GraphPad Prism. Quantiles were determined by ordinary least squares regression using the statsmodels package for Python (Seabold and Perktold 2010). For each centile, the formula ‘telomere_length ~ sqrt_age’ was fit using the quantreg function to determine the gradient and intercept of the line.

## Results

### DC patients exhibit extreme telomere shortening

We undertook STELA analysis of the XpYp telomere on DNA samples derived from unsorted peripheral blood samples obtained from DC patients (*n* = 5), including two EBV transformed lymphoblastoid lines, in conjunction with a panel of unaffected individuals (*n* = 5). This revealed extreme telomere erosion in patients with DC, with some patient samples exhibiting mean telomere lengths of less than 3 kb and telomere length distributions extending to less than 1 kb (Supplementary Fig. 1). The telomere length distributions observed with STELA in these DC patients were as short as those observed using the same technology in several tumour types (Hyatt et al. 2017; Lin et al. 2010; Simpson et al. 2015; Williams et al. 2017). Telomeres within these length ranges can be subjected to fusion with other telomeric and non-telomeric loci and, thus, are a source of genomic instability within tumour cells that drives clonal evolution and progression (Capper et al. 2007; Escudero et al. 2019; Lin et al. 2014; Tankimanova et al. 2012). We, therefore, examined whether telomere fusion could be detected in the same DC patient samples analysed with STELA. We analysed a total of 150,000 diploid genome equivalents for each sample using single-molecule telomere fusion analysis (Capper et al. 2007; Letsolo et al. 2010). Despite analysing a total of 750,000 diploid genome equivalents across the five patient samples, we detected just one telomere fusion event (data not shown). We conclude that whilst telomere lengths can be extremely short in the peripheral blood cells from DC patients, the telomeres are still capable of maintaining an end-capping function and are not subjected to telomere fusion.

### High-throughput single telomere length analysis

**Fig. 1:**
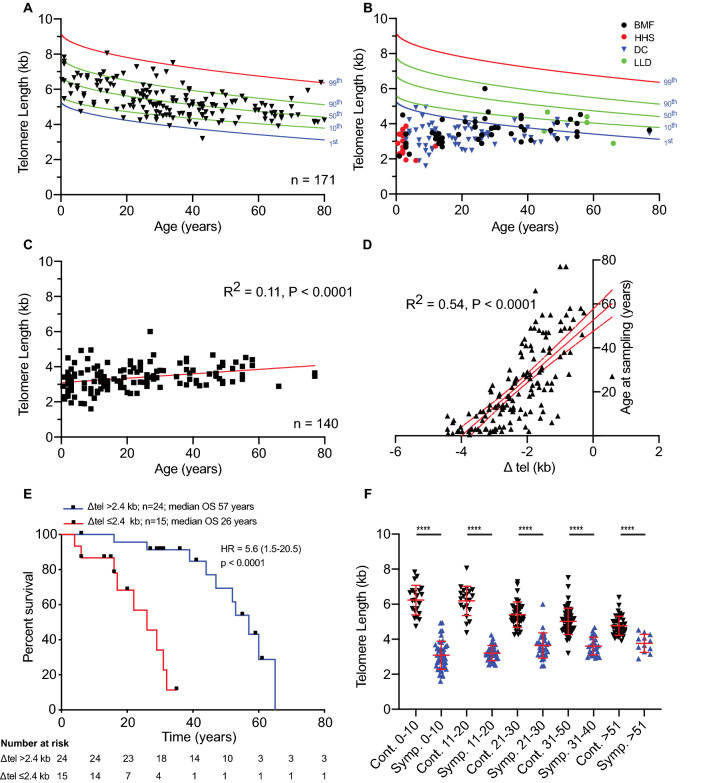
HT-STELA provides diagnostic and prognostic information in telomeropathy patients. **a** Mean 17p telomere length of unaffected individuals plotted as a function of age, together with predicted percentiles. **b** Mean 17p telomere length of symptomatic individuals with defined mutations in genes involved in telomere maintenance by diagnosis plotted together with predicted percentiles of unaffected individuals. *BMF* bone marrow failure, *DC* dyskeratosis congenital, *HHS* Hoyeraal–Hreidarsson syndrome, *LLD* liver and/or lung disease. **c** Mean 17p telomere length of symptomatic individuals with defined mutations in genes involved in telomere maintenance as a function of age. **d** Plotting age-adjusted telomere length from the 50th centile with age (Δ tel). **e** Kaplan Meier curves for overall survival (age at death) in 39 symptomatic patients with defined mutations within genes required for telomere maintenance. *p* values, HR (log-rank) and 95% confidence intervals are indicated on the plots. **f** Telomere length categorised into age ranges as indicated below. Unaffected individuals plotted in black, symptomatic individuals plotted in blue. *p* values (Mann–Whitney) are indicated above (****p < 0.0001), mean and standard deviation are indicated in red

We used HT-STELA at the 17p telomere to define telomere length in DNA derived from peripheral blood samples in a cohort of unaffected control individuals, across the age range from 4 months to 92 years (*n* = 171). Consistent with numerous studies using a range of telomere length analysis technologies (Daniali et al. 2013; Hastie et al. 1990), telomere length declined as a function of age at a rate, defined by linear regression, of 24 bp/year. STELA specifically excludes the telomere-adjacent DNA, it determines only the length of the double-stranded telomere repeat array and does not suffer from the loss of telomere repeat hybridisation signal from shorter telomeres. Thus the telomere length estimates determined with STELA are lower (≅ 3 kb), than those observed with hybridisation-based methodologies such as TRF and flow-FISH (Alder et al. 2018; Baird et al. 2003; Daniali et al. 2013). We applied a linear model with telomere length proportional to the square root of age as a best fit for the data (*R*^2^ = 0.36, *p* > 0.0001, *F* = 96.5), as demonstrated previously, (Alder et al. 2018; Juola et al. 2006) and used this to predict the percentile telomere length distributions based on HT-STELA in the control population (Fig. 1a).

We next used this cohort to determine the measurement error of HT-STELA in peripheral blood samples obtained from the control cohort. Telomere length was determined using HT-STELA on three separate occasions in a panel of 25 samples from the cohort representing the entire measurable range observed. Telomere length measurements generated were used to calculate both intra- and inter-assay coefficient of variation (CV%) across the three assays. As each sample analysed by HT-STELA is tested in triplicate, the intra-assay CV for each measurement can be obtained: each of the three independent HT-STELA assays displayed a low intra-assay CV% (1.9%, 1.4% and 2.3%, respectively; Supplementary Fig. 2). The inter-assay CV across the three assays was similarly low (2.3%). Across the entire control cohort (*n* = 171) the mean intra-assay CV was 1.8%. Thus, HT-STELA provides a fast and accurate system to determine telomere length in peripheral blood DNA samples.

### HT-STELA provides diagnostic and prognostic information in patients with variants in telomere associated genes

We tested whether HT-STELA could be used to discriminate individuals with disease-causing variants in genes related to telomere length maintenance. DNA derived from peripheral blood from a cohort of subjects (*n* = 172) with defined mutations in either *TERT, TERC, DKC1, TINF2, RTEL1* or *ACD* was analysed with HT-STELA (Table 1). The cohort included 32 asymptomatic carriers and 140 symptomatic patients (mean age 22 years) including diagnoses of dyskeratosis congenita (DC, 52.1%), bone marrow failure (BMF, 33.6%), Hoyeraal–Hreidarsson syndrome (HHS, 10.7%) and lung and/or liver disease (LLD, 3.6%; Fig. 1b). Whilst telomere length was positively correlated with age in the symptomatic patients (*R*^2^ = 0.11, *p* < 0.0001; Fig. 1c) this was not observed in the asymptomatic carriers (Supplementary Fig. 3). The positive correlation with age in symptomatic patients was consistent with the decreasing severity of disease as a function of age, (Alder et al. 2018) such that younger individuals tend to have shorter telomeres and more severe disease phenotypes (Fig. 1d). This was apparent in a survival analysis of symptomatic patients where, following optimisation based on Chi-square and hazard ratio (HR, log-rank, Mantel–Cox; Supplementary Fig. 4), those with an age-adjusted telomere length (Δ tel) of < −2.4 kb displayed an inferior survival with a median overall survival of 26 years (HR = 5.6 (1.5–20.5); *p* < 0.0001; *n* = 39) compared to Δ tel > −2.4 kb (median overall survival 57 years; Fig. 1e). In contrast, telomere length without age adjustment was not prognostic (data not shown). This relationship meant that the ability to distinguish patients based on telomere length decreased as function of age (Fig. 1f).

**Fig. 2:**
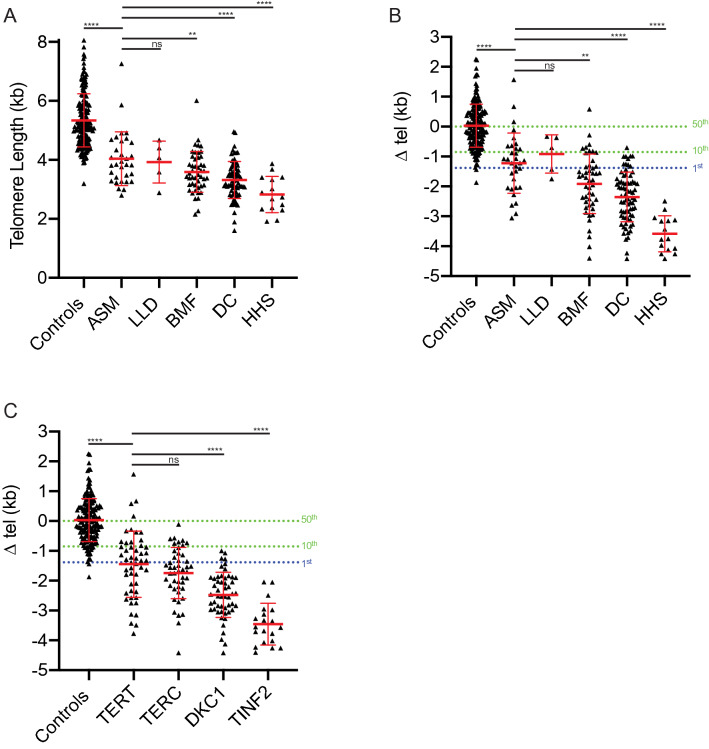
Distinct telomere lengths and age-adjusted telomere lengths is influenced by diagnosis and mutational status. **a** Telomere length distributions broken down by diagnosis. **b** Age-adjusted telomere length differential from the 50th centile (Δ tel) broken down by diagnosis, and **c** by mutational status. *ASM* asymptomatic; other abbreviations as in Fig. 1b. *p* values (Mann–Whitney) are indicated above (****p < 0.0001, ***p < 0.001, **p < 0.01, *ns* not significant), mean and standard deviation are indicated in red

The telomere length distributions observed in the asymptomatic patients were shorter than those observed in control individuals (*p* < 0.0001, Mann–Whitney *U*; Fig. 2a). However in contrast to previous work (Alder et al. 2018), the telomere length of asymptomatic patients was longer than that observed for the symptomatic patients with DC (*p* < 0.0001), HHS (*p* < 0.0001) and BMF (*p* = 0.005), but not for LLD (*p* = 0.35; Fig. 2a), and these differences were more apparent with age-adjusted Δ tel measurement (Fig. 2b). Patients with HHS displayed the most severe telomere length phenotype (Figs. 1d and 2), all HHS patients displayed telomere lengths that were considerably less than the 1st centile of the control population, with a mean telomere length of just 2.8 kb and a Δ tel of −3.6 kb from the age-adjusted 50th centile (Fig. 2a and b). The mean telomere length in the DC patient cohort was 3.3 kb and age-adjusted Δ tel of −2.4 kb from the 50th centile, with 98.6% of the DC patients (72/73) displaying age-adjusted telomere lengths < 10th centile and 86% < 1st centile (63/73). Age-adjusted Δ tel in the BMF patient cohort was more heterogeneous than observed in the DC and HHS cohorts, with age-adjusted Δ tel ranging from 0.58 to -4.4 kb. Consistent with previous reports (Alder et al. 2018; Armanios and Blackburn 2012; Walne et al. 2008) telomere length was clearly dependent on the specific genes mutated: with *TERT* and *TERC* displaying similar age-adjusted mean Δ tel, −1.4 kb and −1.7 kb, respectively (*p* = 0.17 Mann–Whitney *U*), DKC1 −2.5 kb, and with TINF2 displaying the greatest difference of −3.5 kb (Fig. 2c). The telomere length differentials observed between mutated genes accounted for some of the variation in age-adjusted telomere length between the BMF and DC patient cohorts, whilst patients with HHS displayed extensive telomere shortening irrespective of genotype (Supplementary Fig. 5).

### A role for HT-STELA measurement in variant interpretation

In the 134 affected families of the patient cohort described herein, we identified 99 different variants across six different DC-associated disease genes, 31 of which have not been published previously (Supplementary Table 1). Reviewing these variants using the guidelines of the American College of Medical Genetics and Genomics (ACMG) (Kopanos et al. 2019; Richards et al. 2015), we found nearly half were classified as variants of unknown significance (VUS). We then added our HT-STELA data to this interpretation, using the age-adjusted Δ tel as follows: > 99th centile as a strong benign code, > 90th centile as a supporting benign code, < 10th centile as a supporting pathogenic code, < 1st centile as a moderate pathogenic code and Δ tel < -2.4 kb as a strong pathogenic code. This resulted in a significant drop in the number of VUS from 47 to 21, and an increase in the yield of likely pathogenic and pathogenic variants from 50 to 77 (Supplementary Table 1).

### Allelic length distributions detected in DC patients

**Fig. 3:**
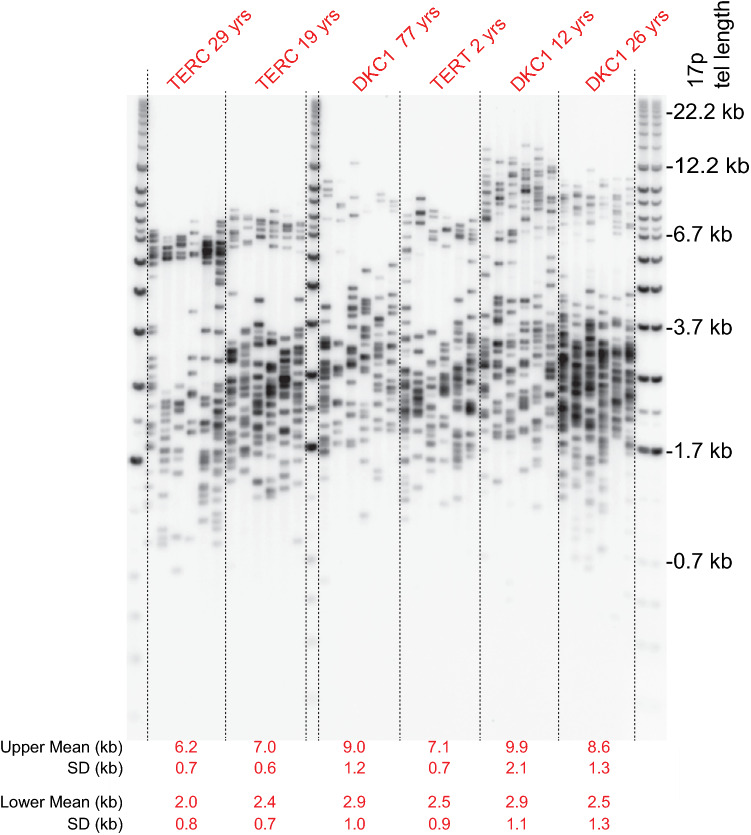
Bimodal telomere length distributions in peripheral blood DNA from telomeropathy patients. Single telomere length analysis (17p) in six patients, the mutated genes are indicated above. The mean and standard deviation (SD) of the telomere length of each modal distribution is detailed below

STELA reveals telomere length distributions at single chromosome ends. In clonal populations, in the absence of telomerase, it is possible to observe bimodal telomere length distributions that arise from allelic telomere length variation (Baird et al. 2003; Britt-Compton et al. 2006). Using HT-STELA, we observed three individuals in our cohort of unaffected individuals (*n* = 171) that displayed bimodal telomere length distributions in their peripheral blood samples (1.7%). In contrast, we observed 25 bimodal telomere length distributions within the patient cohort (*n* = 172; 15%; *p*-value 1.3 × 10^–5^, Fisher exact test). We verified bimodality at the 17p telomere using single-molecule STELA (Britt-Compton et al. 2006) and revealed differences in length of up to 6.96 kb between the distributions (Fig. 3). Whilst the bimodal telomere length distributions could be consistent with allelic telomere length variation, such differentials can also occur as a consequence of cellular heterogeneity within sub-populations of cells exhibiting distinct replicative histories. To distinguish between these possibilities, we took advantage of our ability to analyse allele-specific telomere length distributions at the XpYp telomere. This works by utilising telomere-adjacent single nucleotide polymorphism (SNP) to specifically analyse telomeric alleles in heterozygous individuals (Baird et al. 2003). We used HT-STELA to analyse 50 DC samples at the XpYp telomere using HT-STELA to detect individuals displaying bimodal distributions. Bimodal XpYp telomere length distributions were observed in 6 samples (12%) and of these 6 samples only 2 showed bimodality at both XpYp and 17p telomeres; thus, most examples of bimodality were exclusive to one of the telomeres analysed. We performed allele-specific STELA (Baird et al. 2003) at XpYp for the samples which showed clear bimodal telomere length distributions at XpYp. Five of these were heterozygous at the XpYp telomere-adjacent SNPs allowing us to independently amplify each telomeric allele. This revealed clear allele-specific telomere length distributions for all five samples (Supplementary Fig. 6), the most striking difference being in samples 25 and 51 with inter-allelic differences in mean telomere length of 3.7 and 4.1 kb, respectively. These results mirror the bimodality seen in HT-STELA and point to inter-allelic differences as being the source of the bimodal telomere length distributions observed in DC patients.

## Discussion

The differential diagnosis of patients presenting with bone marrow failure can be challenging. In some cases, disease features are pathognomonic but more often than not additional functional or genetic testing is warranted. While hypersensitivity to clastogens is a well-established and specific functional test for the Fanconi anaemia subgroup of patients, telomere length testing is now becoming a part of the routine work-up of bone marrow failure patients, to investigate the possibility of a telomeropathy. Awareness is also growing of the extent to which short telomeres impact on health issues beyond the realm of haematology, particularly in lung and/or liver disease. Currently, there are two procedures in common use, flow-FISH (Baerlocher et al. 2006) and quantitative PCR (Cawthon 2002). While the former is regarded as the gold standard, this procedure requires primary peripheral blood cells, is relatively expensive, and requires a calibration step to give a kb measurement. The quantitative PCR measurement is popular as it is relatively easy to set up but shows a considerable inter-assay variance and requires a calibrator to give a length measurement. Here, we show that HT-STELA provides a robust alternative, as a rapid DNA-based test that gives a direct measurement of telomere length in kb with a low measurement error.

We show that HT-STELA both performs well in discriminating a defined group of patients with dyskeratosis congenita and related disorders and provides important prognostic information. Access to the relatively large cohort described in this study, followed up in a single centre, has enabled us to show the highly significant hazard ratio (HR = 5.6) for survival when patients are stratified on the basis of a Δ tel above or below −2.4 kb, indicating that patients with mean telomere lengths below this threshold have over a fivefold greater risk of death. We believe this is the first time that the extent of telomere shortening has been shown to have such a significant impact on life expectancy.

The data presented here reveal that telomeropathy patients can exhibit telomere distributions that are shorter than previously considered, with mean telomere length distributions below 3 kb frequently detected in patients across a range of ages, but more frequently in patients < 20 years. These short telomere length distributions are within the length ranges observed using the same technology in cells undergoing replicative senescence (Baird et al. 2003; Britt-Compton et al. 2006) or a telomere-driven crisis (Capper et al. 2007) in vitro, or indeed within haematological malignancies in vivo (Hyatt et al. 2017; Lin et al. 2014), in which telomere fusion is readily detected. Despite this, we were unable to detect evidence for telomere dysfunction in the form of telomere fusion events, implying that these telomeres may not be capable of driving the same kind of genomic instability as seen in cancer and crisis. However cells with telomeres in these short length ranges, with limiting telomerase activity, are likely to exhibit a limited replicative lifespan and functional activity (Valenzuela and Effros 2002).

We observed bimodal distributions consistent with allelic variation in the patient cohort that were rare in unaffected individuals. In cells that express telomerase, telomere length heterogeneity arises from a combination of end-replication losses and processing, coupled with telomerase mediated telomeric elongation (Britt-Compton et al. 2009; Levy et al. 1992). This means that the length distributions of individual telomeric alleles overlap and that allelic variation can be difficult to discern. Telomerase expressing cancer derived cell lines typically display heterogenous telomere length profiles, with no evidence of bimodality (Capper et al. 2007). However, when telomerase activity is abrogated and clonal populations are derived, the clonal derivatives uniformly display bimodal telomere length distributions; whereas, control clonal population expressing telomerase do not (Jones et al. 2014). We, therefore, consider the observation of bimodal telomere length variation in the patient cohort as a manifestation of reduced levels of functional telomerase activity in these individuals.

Short telomeres are unlikely to be variant specific. However, a telomere length measurement, in a patient who also has a novel variant of unknown significance (VUS) in one of the known disease genes, may influence the interpretation of that variant. For example, if the telomere length in a patient with such a VUS is long enough to be in the normal range, it might suggest that the variant is benign. Conversely, the presence of very short telomeres may influence the interpretation in favour of pathogenicity. In our extensively characterised cohort, we found nearly half of the variants would be classified as VUS using the ACMG criteria (in the absence of a supporting telomere measurement). This is not unusual for private missense variants in known disease-associated genes (particularly in the *TERT* gene, where symptoms may not manifest until adulthood) allowing disease-causing variants to exist in the healthy population at low frequency. In implementing such a stratified approach, we find that the proportion of VUS declines to ~ 20%, which we believe is likely to be a more realistic assessment of the variants identified in this patient cohort. Together, this suggests the use of HT-STELA, weighted according to its position with respect to the normal range, provides added value in variant interpretation.

Here, we have demonstrated that HT-STELA provides a rapid and high-resolution approach to determine telomere length distributions directly from peripheral blood DNA samples. The telomere length distribution over the age range of the human population and the rate of telomere erosion is similar to that observed with other methodologies. However, as STELA is not limited by a lower length threshold of detection, it is capable of detecting telomeres in the shorter length ranges. Like flow-FISH, HT-STELA displays a low measurement error and, thus, has the potential to be applied for clinical diagnostic and prognostic applications. However, as HT-STELA is DNA based, it can be applied to a broader range of sample types and does not require that cells are stored in viable state for flow cytometry. We anticipate these reduced sample handling requirements will support scalability whilst minimising pre-analytical variability. Moreover, the data generated in this study reveal that the analysis of specific immune cell sub-sets is not required; instead, analysis of whole peripheral blood leukocyte DNA is sufficient to provide a high-level of discrimination between mutation carriers and the control population. In conclusion, HT-STELA represents a new high-quality and user-friendly laboratory test for diagnosing patients with bone marrow failure or other abnormalities arising from an underlying telomeropathy.

## Supplementary Information

Below is the link to the electronic supplementary material.

Supplementary file1 (PDF 2193 KB)
