# Supplementary material for: High-throughput STELA provides a rapid test for the diagnosis of telomere biology disorders
Source: Hum Genet. 2021 Mar 11;140(6):945–55. doi: 10.1007/s00439-021-02257-4 (PMC8099822; doi:10.1007/s00439-021-02257-4)
Supplement: Supplementary file 1 — Supplementary file1 (PDF 2193 KB) [file 439_2021_2257_MOESM1_ESM.pdf]

Supplemental data

High-throughput STELA provides a rapid test for the diagnosis of telomere biology disorders

Kevin Norris[1], Amanda J. Walne[2], Mark J. Ponsford[3,4], Kez Cleal[1], Julia W. Grimstead[1], Alicia Ellison[2], Jenna Alnajar[2], Inderjeet Dokal[2], Tom Vulliamy[2¶] and Duncan M. Baird[1¶]

[1] Division of Cancer and Genetics, School of Medicine, Cardiff University, Heath Park, Cardiff, CF14 4XN, UK.

[2]Centre for Genomics and Child Health, Blizard Institute, Barts and The London School of Medicine and Dentistry, Queen Mary University of London, London, E1 2AT, UK.

[3] Immunodeficiency Centre for Wales, University Hospital of Wales, Heath Park, Cardiff CF14 4XW, UK.

[4] Division of Infection, Inflammation and Immunity, School of Medicine, Cardiff University, Heath Park, Cardiff, CF14 4XN, UK.

¶joint senior authors

*Correspondence: email:bairddm@cardiff.ac.uk; t.vulliamy@qmul.ac.uk;

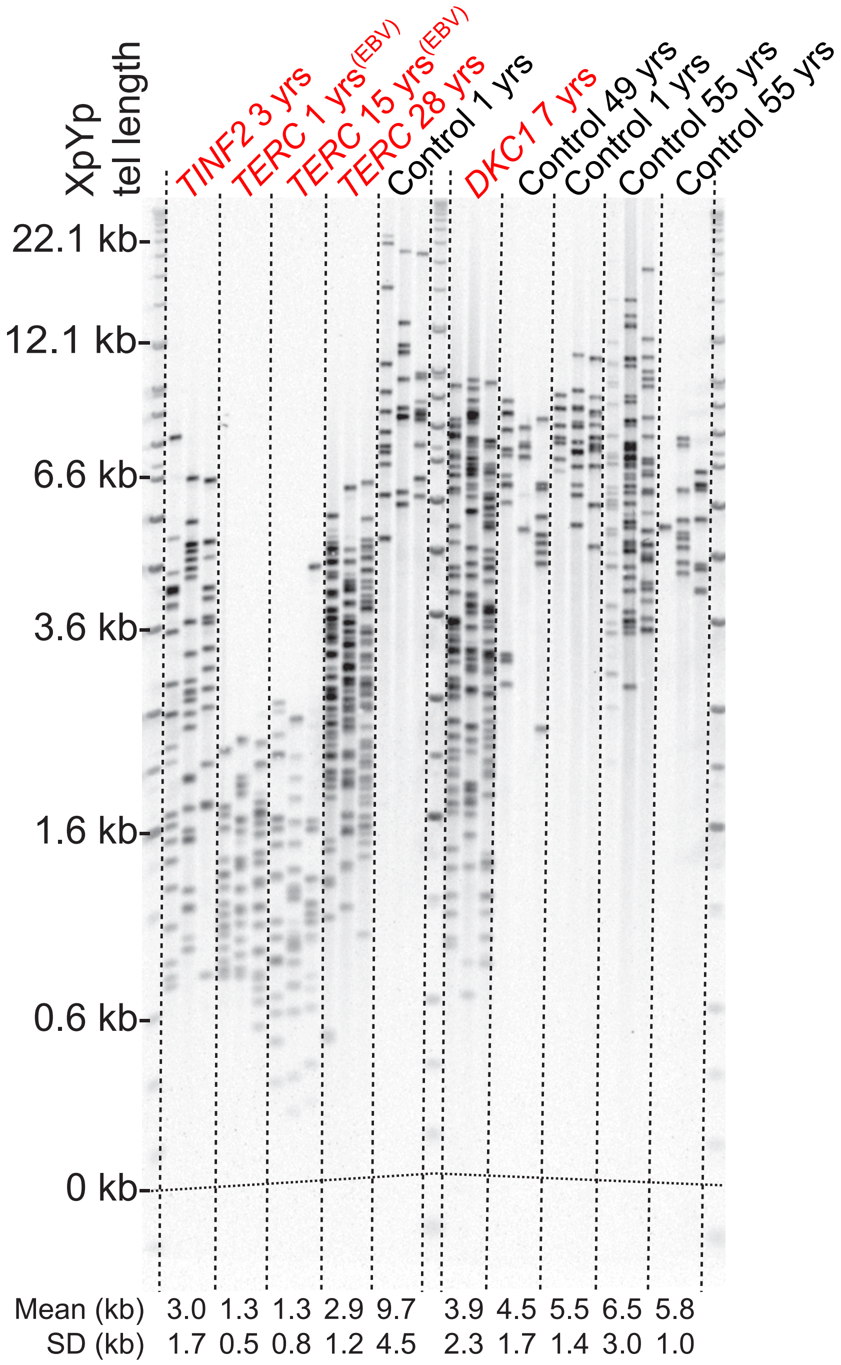

Supplementary figure 1. STELA reveals extreme telomere shortening in patients with telomeropathies. XpYp STELA gel showing 5 DC patients (red) and 5 healthy controls (black). Mutated genes are indicated above with mean and standard deviation (SD) of telomere length distributions displayed below; samples obtained from EBV transformed lymphoblastoid cell lines are indicated.

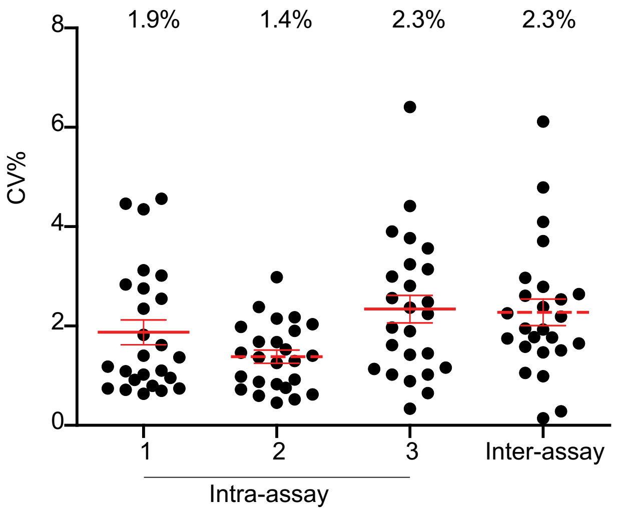

Supplementary figure 2. HT-STELA provides an accurate system to determine telomere length in peripheral blood DNA samples. Samples from 25 unaffected individuals analysed independently three times, each time in triplicate. Scatter plots depict intra-assay CV for the three independent analyses and the inter-assay CV as indicated.

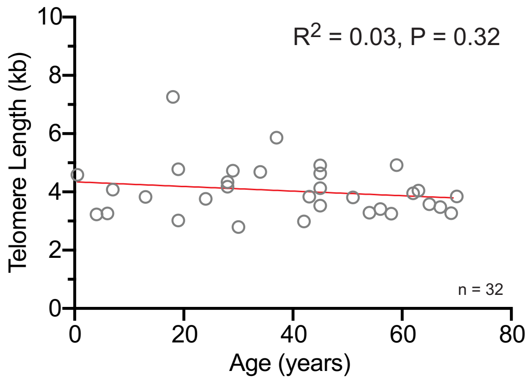

Supplementary figure 3. Mean telomere length of asymptomatic individuals with defined mutations in genes involved in telomere maintenance as a function of age.

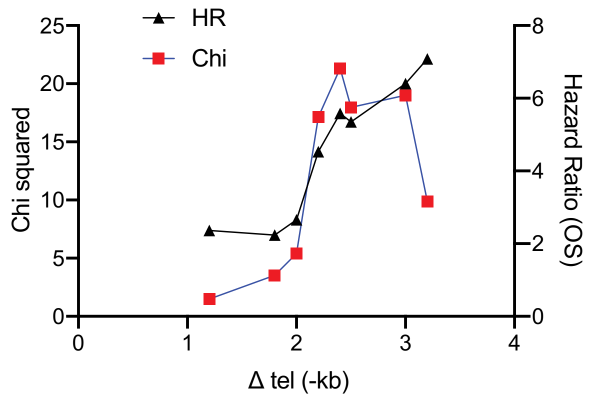

Supplementary figure 4. Optimising telomere length cut-off for patient stratification. Kaplan-Meier analysis for overall survival was undertaken at multiple telomere length cut-offs, Chi-square (red squares, left axis) and HR (Black triangles, right axis; Log-rank, Mantel-Cox) were plotted as a function of age-adjusted Δ tel.

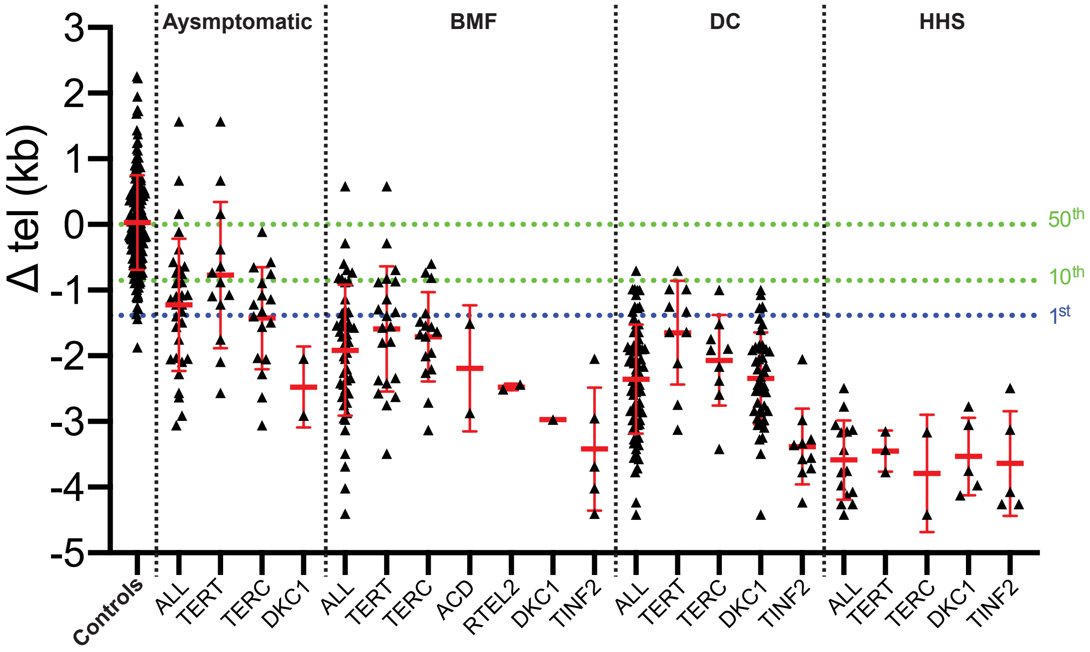

Supplementary figure 5. Telomere length heterogeneity within diagnostic sub-groups is partially accounted for by differences between mutated genes. Scatter plots depicting telomere length of symptomatic and asymptomatic patients broken down by mutational status.

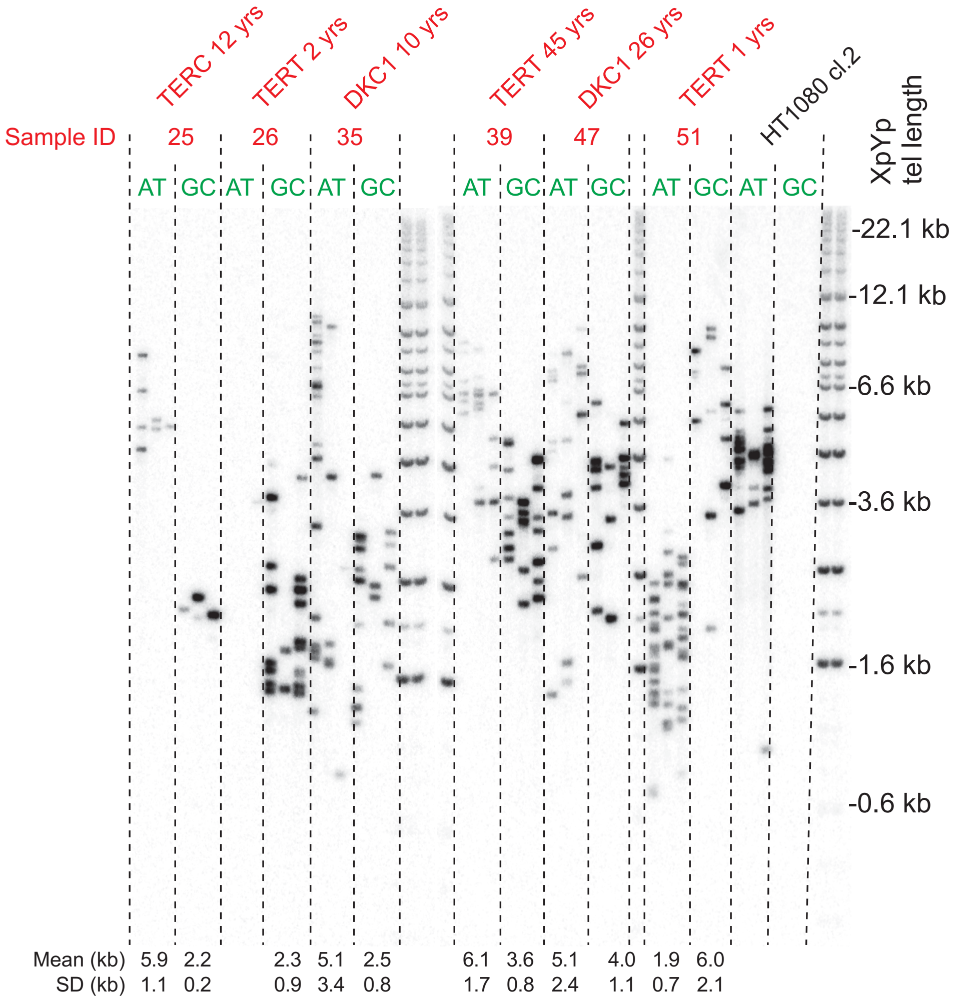

Supplementary figure 6. Allelic telomere length variation accounts for bimodal telomere length distributions in telomeropathy patients. Allele-specific single telomere length analysis (STELA). Six individuals with defined mutations, as indicated above, each individual is heterozygous for telomere-adjacent single nucleotide polymorphisms (AT and GC). Allele-specific STELA was performed using oligonucleotides specific to these polymorphic sites. Mean and standard deviation (SD) of each distribution is indicated below.

Gene	Sequence change	Amino acid substitution	PubMed ID	ACMG Codes	Variant status	Δ tel (sq root age)	TL Code	Final status
ACD	c.284T>A	p.Lys95Gln	30064976	PM2, BP1, BP4	LB	-2.87	TL_PS	VUS
ACD	c.508_510del	p.Lys170del	25205116	PM2, PM4	VUS	-1.51	TL_PP	VUS
DKC1	c.113T>C	p.Ile38Thr	12437656	PM2, PP3, PP5, PS4_moderate	LP	-3.75	TL_PS	P
DKC1	c.119C>G	p.Pro40Arg	9590285	PM1, PM2, PP3, PP5, PS4_supporting, PP1_strong	P	-2.53	TL_PS	P
DKC1	c.121G>A	p.Glu41Lys	10364516	PM2, PP3, PP5, PS4_supporting	VUS	-2.20	TL_PM	LP
DKC1	c.146C>T	p.Thr49Met	10583221	PM2, PP3, PP5, PM6, PS4	P	-2.77	TL_PS	P
DKC1	c.194G>C	p.Arg65Thr	10364516	PM2, PP3, PP5	VUS	-2.55	TL_PS	LP
DKC1	c.200C>T	p.Thr67Ile	16332973	PM2, PP3, PS4_supporting	VUS	-2.52	TL_PS	LP
DKC1	c.204C>A	p.His68Gln	16332973	PM2, PP3, PM5, PS4_supporting	LP	-3.97	TL_PS	P
DKC1	c.230A>G	p.Asn77Ser	.	PM2, PP3	VUS	-1.00	TL_PP	VUS
DKC1	c.1049T>C	p.Met350Thr	10364516	PM1, PM2, PM5, PP2, PP3, PP5, PS4_supporting	LP	-1.90	TL_PM	LP
DKC1	c.1058C>T	p.Ala353Val	10364516	PM1, PM2, PP3, PP5, PS4, PM6	P	-2.46	TL_PS	P
DKC1	c.1151C>T	p.Pro384Leu	11379875	PM2, PP3	VUS	-2.86	TL_PS	LP
DKC1	c.1176G>C	p.Met392Ile	.	PM2, PP3	VUS	-2.76	TL_PS	LP
DKC1	c.1204G>A	p.Gly402Arg	11379875	PM2, PP3, PP5	VUS	-2.02	TL_PM	LP
DKC1	c.1205G>A	p.Gly402Glu	9590285	PM2, PP3, PP5, PS4_supporting, PP1_moderate	LP	-1.89	TL_PM	LP
DKC1	c.1223C>T	p.Thr408Ile	16332973	PM2, PP3, PP5, PS4_moderate	LP	-2.24	TL_PM	LP
DKC1	c.1232_1243dupCCTGGAAGCAGG	p.Gln414_Glu415insAlaTrpLysGln	.	PM2, PM4	VUS	-2.97	TL_PS	LP
DKC1	c.1258_1259delAGinsTA	p.Ser420Tyr	16332973	PM2	VUS	-3.09	TL_PS	LP
RTEL1	c.3286G>T	p.Gly1096Trp	28495916	PM2, PP3	VUS	-2.51	TL_PS	LP
TERC	n.-2664_316del	.	15098033	PVS1, PM2	LP	-3.05	TL_PS	P
TERC	n.35C>T	.	18931339	.	VUS	-0.60	TL_N	VUS
TERC	n.36C>T	.	21931702	PM2	VUS	-2.38	TL_PM	VUS
TERC	n.53_87del	.	17640862	PVS1, PM2, PS3	P	-2.00	TL_PM	P
TERC	n.67G>A	.	21931702	PM2, PS3	LP	-1.48	TL_PP	LP
TERC	n.72C>G	.	12090986	PM2, PS3, PS4_supporting	LP	-1.35	TL_PP	LP
TERC	n.83T>G	.	21931702	PM2	VUS	-1.67	TL_PM	VUS
TERC	n.93G>C	.	.	.	VUS	-2.71	TL_PS	VUS
TERC	n.95_96delGC	.	21931702	PM2, PP3, PM4	VUS	-1.32	TL_PP	LP
TERC	n.95G>C	.	26024875	PM2, PP3, PS3	LP	-3.06	TL_PS	P
TERC	n.96_97delCT	.	15098033	PM2, PP3, PS3	LP	-2.16	TL_PM	LP
TERC	n.107_108delinsAG	.	11574891	PM2, PP3, PS3	LP	-1.74	TL_PM	LP
TERC	n.110_113delGACT	.	12090986	PM2, PP3, PP1, PS3, PS4_moderate	LP	-1.14	TL_PP	LP
TERC	n.126A>G	.	21931702	PM2, PP3, PS3	LP	-1.63	TL_PM	LP
TERC	n.129_140delinsGT	.	.	PVS1, PM2	LP	-2.20	TL_PM	P
TERC	n.149_156delCACCGTTC	.	.	PVS1, PM2	LP	-1.70	TL_PM	P
TERC	n.180C>T	.	17640862	PM2, PP3	VUS	-1.56	TL_PP	VUS
TERC	n.182G>A	.	21931702	PM2, PP3, PS3	LP	-2.26	TL_PM	LP
TERC	n.183C>G	.	.	PM2, PP3	VUS	-0.70	TL_N	VUS
TERC	n.202T>G	.	26024875	PM2	VUS	-1.89	TL_PM	VUS
TERC	n.212C>G	.	19760794	PM2, PS3, PS4_supporting	LP	-1.00	TL_PP	LP
TERC	n.213T>A	.	.	PM2	VUS	-3.13	TL_PS	LP
TERC	n.242C>T	.	21931702	PM2	VUS	-4.42	TL_PS	LP
TERC	n.287C>G	.	21931702	PM2, PS3	LP	-1.56	TL_PP	LP
TERC	n.321C>A	.	.	PM2, PP3	VUS	-2.59	TL_PS	LP
TERC	n.323_336delinsAGACCC	.	.	PVS1, PM2, PP3	P	-1.52	TL_PP	P
TERC	n.377A>G	.	21931702	PM2, PP3, PS3	LP	-1.08	TL_PP	LP
TERC	n.378_451del	.	11574891	PVS1, PM2, PP1_moderate	P	-2.03	TL_PM	P
TERC	n.408C>G	.	11574891	PM2, PS3	LP	-2.05	TL_PM	LP
TERC	n.448A>G	.	26024875	PM2, PS3	LP	-3.16	TL_PS	P
TERT	c.166G>C	p.Val56Leu	21931702	PM2, PP3, PS3	LP	-2.09	TL_PM	LP
TERT	c.248G>C	p.Arg83Pro	21931702	PM2, PP3	VUS	-2.57	TL_PS	LP
TERT	c.355G>A	p.Val119Met	26024875	PM2	VUS	-1.64	TL_PM	VUS
TERT	c.1381G>T	p.Val461Leu	.	PM2, PP3	VUS	-3.77	TL_PS	LP
TERT	c.1386delC	p.Tyr462Ter	.	PVS1, PM2	LP	-3.15	TL_PS	P
TERT	c.1396C>T	p.Arg466Trp	.	PM2, PP3	VUS	-3.43	TL_PS	LP
TERT	c.1445delA	p.His482ProfsTer27	.	PVS1, PM2, PP3, PP1	P	-1.07	TL_PP	P
TERT	c.1570C>G	p.Pro524Ala	26024875	PM2, PP3, PS3	LP	-2.75	TL_PS	P
TERT	c.1710G>C	p.Lys570Asn	16990594	PM2, PP3, PS4_supporting	VUS	-1.14	TL_PP	LP
TERT	c.1769+1G>A	n/a	22664374	PVS1, PM2, PP3	P	-0.69	TL_N	P
TERT	c.1805C>T	p.Ser602Leu	.	PP3, PS4_supporting	VUS	-3.15	TL_PS	LP
TERT	c.1892G>A	p.Arg631Gln	18460650	PM2, PP3, PP5, PS3, PS4, PP1_strong	P	-1.09	TL_PP	P
TERT	c.1990G>A	p.Val664Met	.	PM2	VUS	-0.33	TL_N	VUS
TERT	c.2078T>G	p.Phe693Cys	.	PM2, PP3	VUS	-0.88	TL_PP	VUS
TERT	c.2124T>G	p.Phe708Leu	.	PM2, PP3	VUS	-0.82	TL_N	VUS
TERT	c.2146G>A	p.Ala716Thr	.	PM2, PP3, PP5	VUS	-1.75	TL_PM	LP
TERT	c.2152G>A	p.Asp718Asn	21931702	PM2, PP3	VUS	-0.98	TL_PP	LP
TERT	c.2173C>T	p.Leu725Phe	26024875	PM2, PP3	VUS	-2.37	TL_PM	LP
TERT	c.2227C>T	p.Arg743Trp	.	PP3, PP5, PS4_supporting	VUS	-1.57	TL_PM	LP
TERT	c.2255A>G	p.His752Arg	.	BS2, BP4, PS4_moderate	LB	0.58	TL_N	LB
TERT	c.2312C>G	p.Pro771Arg	.	PM2, PP3	VUS	-2.42	TL_PS	LP
TERT	c.2318T>C	p.Met773Thr	26024875	PM2, PP3	VUS	-1.55	TL_PP	VUS
TERT	c.2329G>T	p.Val777Leu	26024875	PM2, BP4, PS4_supporting	VUS	-1.43	TL_PP	VUS
TERT	c.2354C>T	p.Pro785Leu	19760794	PM2, BP4, PS3	LP	0.67	TL_N	LP
TERT	c.2431C>T	p.Arg811Cys	17785587	PP3, PP5, PS4_supporting	VUS	-1.91	TL_PM	LP
TERT	c.2494C>T	p.Pro832Ser	.	PM2, PP3	VUS	-3.49	TL_PS	LP
TERT	c.2529C>G	p.Ser843Arg	.	PM2, PP3	VUS	-0.86	TL_N	VUS
TERT	c.2581C>T	p.Gly861Arg	21931702	PM2, PP3, PS3	LP	-1.39	TL_PP	LP
TERT	c.2594G>A	p.Arg865His	17460043	PP3, PP5, PS4	LP	-1.32	TL_PP	LP
TERT	c.2599G>T	p.Val867Leu	.	PP3, PM5	VUS	-3.12	TL_PS	LP
TERT	c.2750C>T	p.Thr917Met	.	BS2	VUS	-0.37	TL_N	VUS
TERT	c.2828A>C	p.Gln943Pro	.	PM2, BP4	VUS	-1.33	TL_PP	VUS
TERT	c.2836T>C	p.Tyr946His	.	PM2, PP3	VUS	-1.29	TL_PP	VUS
TERT	c.3082A>C	p.Asn1028His	21931702	PM2, PP3, PS3	LP	-2.11	TL_PM	LP
TERT	c.3187G>A	p.Gly1063Ser	21931702	PM2, PP3, PP1_strong, PS4_moderate	LP	-1.25	TL_PP	LP
TERT	c.3230C>T	p.Ala1077Val	.	PM2, PP3	VUS	-0.98	TL_PP	VUS
TINF2	c.805C>T	p.Gln269Ter	21477109	PVS1, PM2, PM6, PS4_supporting	P	-3.68	TL_PS	P
TINF2	c.811C>T	p.Gln271Ter	21477109	PVS1, PM2, PS4_supporting	P	-4.26	TL_PS	P
TINF2	c.815G>A	p.Trp272Ter	.	PVS1, PM2, PM6	P	-3.36	TL_PS	P
TINF2	c.826delA	p.Arg276GlyfsTer41	21199492	PVS1, PM2, PM6	P	-3.71	TL_PS	P
TINF2	c.838A>T	p.Lys280Ter	18669893	PVS1, PM2, PP3, PM6, PS4_supporting	P	-4.26	TL_PS	P
TINF2	c.844C>T	p.Arg282Cys	18669893	PS4, PM1, PM2, PM5, PP3, PP5, PM6	P	-3.55	TL_PS	P
TINF2	c.845G>A	p.Arg282His	18252230	PS4, PM1, PM2, PM5, PP3, PP5	P	-2.49	TL_PS	P
TINF2	c.845G>T	p.Arg282Leu	.	PM1, PM2, PM5, PP3	LP	-2.04	TL_PM	LP
TINF2	c.847C>T	p.Pro283Ser	18669893	PM2, PP3, PP5, PM6, PS4_moderate	LP	-2.98	TL_PS	P
TINF2	c.849delC	p.Thr284GlnfsTer33	21931702	PVS1, PM2	LP	-2.95	TL_PS	P
TINF2	c.857delTinsGC	p.Met286SerfsTer5	21199492	PVS1, PM2, PM6	P	-3.57	TL_PS	P
TINF2	c.860T>C	p.Leu287Pro	18669893	PM2, PP3	VUS	-3.77	TL_PS	LP
TINF2	c.867_868insC	p.Phe290LeufsTer2	18669893	PVS1, PM2, PS4_supporting	P	-4.23	TL_PS	P

Supplementary table 1. Curation of the 99 variants identified in affected individuals. American College of Medical Genetics and Genomics (ACMG) criteria were applied. LB = likely benign, LP = likely pathogenic and P = pathogenic. The PS4 and PP1 codes were modified in line with recommendations recently described for the curation of variants in RUNX1(Luo et al. 2019) as follows: PS4: ≥ 4 previously reported probands; PS4_moderate: 2-3 previously reported probands; PS4_supporting: 1 previously reported proband; PP1: segregating in 3 or 4 meioses; PP1_moderate: segregating in 5 or 6 meioses; PP1_strong: segregating in 7 or more meioses. The PS3 code was applied where a TRAP assay for telomerase activity has shown <20% wild type activity. Telomere lengths (TL) were added to the curation as follows: TL_neutral (TL_N): Δ tel between the 10th and 90th centiles of the normal range (-0.86 to 0.89 kb); TL_pathogenic_supporting (TL_PP): Δ tel < 10th centile of the normal range (< -0.86 kb); TL_pathogenic_moderate (TL_PM): Δ tel < 1st centile of the normal range (< -1.56 kb); TL_pathogenic_strong (TL_PS): Δ tel < -2.4kb.
